# Pediatrics

**Published:** 1902-04

**Authors:** Maud J. Frye

**Affiliations:** Buffalo, N. Y.


### Pediatrics.

CONDUCTED BY MAUD J. FRYE, M. D., Buffalo, N. Y.

### ULCERO MEMBRANOUS ANGINA.

SOBEL and HERRMAN (*New York Medical Journal*, December, 1901,) call attention to an infrequently recognised, though not uncommon form of sore throat, and report twelve cases in children. Microscopically these cases show the same fusiform bacilli and spirilla which are found in ulcerative stomatitis.

In the twelve cases reported the ulceration was with one exception limited to the tonsils, usually to one tonsil. It varied in size, sometimes involving the greater part of the tonsil, was irregularly oval in shape and chancroidal in appearance. A swab applied to the lesion enters a cavity one-eighth to one-half inch in depth. The color is either yellowish, greenish-gray or a dirty-light brown. There is some elevation of temperature, although constitutional symptoms are usually wanting. The complaint is of sore throat and pain on deglutition. The submaxillary glands on the side of the lesion are involved.

It differs clinically from diphtheria in being distinctly an ulcerative process with no tendency to spread beyond the tonsil, and in the absence of the usual constitutional symptoms of diphtheria. The mild constitutional symptoms and the appearance of the tonsils distinguish it from follicular tonsillitis. Smears from the lesion stained show the characteristic bacilli and spirilla. Cultures are chiefly useful in proving the absence of the Klebs-Löffler bacillus.

The prognosis is invariably good. Treatment consists in local applications made daily, silver nitrate 3 to 5 per cent. being oftenest used. The patients were cured in from 6 to 26 days. Diphtheria antitoxin, which was twice given, had no effect on the disease.

---

### DIPHTHERIA COMPLICATING MEASLES.

BLAKELY and BURROWS (*Boston Medical and Surgical Journal*, July 25, 1901,) call attention to the frequency with which diphtheria complicates measles. In a series of 157 cases in which the diseases were combined, 54 deaths occurred, or 34 per cent., the death-rate during the same period for uncomplicated cases of diphtheria being 13 per cent. Of this whole number 82 had laryngeal diphtheria and of these 36 died. From their experience the authors make the following deductions: The existence of diphtheria or the possibility of its onset should be considered in every case of measles, for the congestion of the mucous membrane of the tonsils and air-passages caused by the measles process renders it especially vulnerable, an unusually good field for the growth of the bacilli of diphtheria. Nasal obstruction or laryngeal obstruction arising during an attack of measles almost certainly means diphtheria. If the initial fever of measles diappears, and there is later a sudden rise of temperature, or if the cough becomes "brassy" in quality or paroxysmal in character and is accompanied by an elevated temperature, the possibility of measles must be considered. If the initial fever persists and aphonia develops, diphtheria is probably the cause. Uncomplicated measles is usually accompanied by a more or less abundant serous nasal discharge; but if this discharge becomes purulent or muco-purulent in character, or if there is partial or complete nasal obstruction accompanied by a glairy discharge, diphtheria should be suspected. In all obscure cases the patient should be given antitoxin. The earlier in the course of measles diphtheria develops the more serious is the outlook.

---

### OTITIS MEDIA NEONATORUM.

JOACHIM (*New Orleans Medical and Surgical Journal*) calls attention to the frequency with which this affection occurs in early infancy. He makes a plea for its more frequent recognition. The most essential symptoms are restlessness, increased temperature and loss of weight. An involuntary persistent throwing about of the head; a continual whimpering with occasional loud cries of pain, often taken for colic; the refusal of food, due to pain, caused by deglutition and consequent loss of weight; and the often observable reaching for the ear by the infant, or the loud cries when the ear is touched, should direct attention and investigation to the probable existence of acute middle-ear inflammation. Diarrhea, uncontrollable by properly regulated diet and treatment, meningeal symptoms and convulsions, are often in the train of symptoms of acute infantile middle-ear disease. Ear inflammation in the course of the acute exanthemata and broncho-pneumonia should be watched for.

### MILK MODIFICATION.

McDonald (*Boston Medical and Surgical Journal*, November 21, 1901,) calls attention to a point usually overlooked in milk modification. Human milk contains caseinogen and lactalbumin in equal proportions, while in cow's milk there is but one part of lactalbumin to six parts of caseinogen. In the ordinary process of modification the lactalbumin is still further reduced. Another marked difference between human and cow's milk is in the condition of the phosphorus which both contain. In the former it exists in organic, in the latter in inorganic compounds. He suggests that both these deficiencies may be somewhat overcome by the use of whole egg, together with cereal, along with the present method of milk modification.

